# Factors Underlying Racial Disparities in Sepsis Management

**DOI:** 10.3390/healthcare6040133

**Published:** 2018-11-19

**Authors:** Matthew DiMeglio, John Dubensky, Samuel Schadt, Rashmika Potdar, Krzysztof Laudanski

**Affiliations:** 1 Philadelphia College of Osteopathic Medicine, Philadelphia, PA 19131, USA; johndub@pcom.edu (J.D.); samuelsc@pcom.edu (S.S.); 2 Department of Anesthesiology and Critical Care, Hospital of the University of Pennsylvania, Philadelphia, PA 19104, USA; 3 Department of Hematology & Oncology, Albert Einstein Health Network, Philadelphia, PA 19141, USA; PotdarRa@einstein.edu; 4 Leonard Davis Institute for Healthcare, Philadelphia, PA 19104, USA

**Keywords:** sepsis, racial disparities, critical illness

## Abstract

Sepsis, a syndrome characterized by systemic inflammation during infection, continues to be one of the most common causes of patient mortality in hospitals across the United States. While standardized treatment protocols have been implemented, a wide variability in clinical outcomes persists across racial groups. Specifically, black and Hispanic populations are frequently associated with higher rates of morbidity and mortality in sepsis compared to the white population. While this is often attributed to systemic bias against minority groups, a growing body of literature has found patient, community, and hospital-based factors to be driving racial differences. In this article, we provide a focused review on some of the factors driving racial disparities in sepsis. We also suggest potential interventions aimed at reducing health disparities in the prevention, early identification, and clinical management of sepsis.

## 1. Introduction

Sepsis, a syndrome characterized life threatening organ dysfunction secondary to infection, is one of the most common causes of inpatient mortality [1,2]. In the United States, sepsis affects approximately 1.7 million patients annually and contributes to 265,000 deaths each year [3]. The economic burden related to sepsis management is significant with approximately 38 billion USD spent annually in inpatient costs [4]. The excess charges related to readmission and increased healthcare utilization following the initial event are significant [5,6,7]. Current efforts are focused on early detection and protocol-driven management or “sepsis bundles” [8,9]. These bundles include interventions such as administration of resuscitative fluids, antibiotics, and vasoactive medications to maintain physiologic parameters at recommended levels. They should also be completed within discrete time frames (such as three- and six-hour bundles), since prompt management is critical [9]. Bundle target adherence has been associated with decreased in-hospital mortality and thus is regarded as the standard of care in sepsis management [10].

Nonetheless, wide variability in mortality rates persist despite standardization of care. This suggests the presence of healthcare disparity, which is defined as the “differences in the quality of health care that are not due to access-related factors or clinical needs, preferences and appropriateness of intervention” [11]. Disparities based on factors such as gender, socioeconomic status, and race are found across the spectrum of healthcare [11,12,13]. In regards to infection and sepsis, minority populations have a higher adjusted incidence, hospitalizations, complications, and deviations from standard care compared to white populations [4,14,15,16,17].

While the presence of racial disparities in the management of sepsis has been commonly established in the literature, recent studies have produced seemingly contradictory results. Since minority race is a marker for poverty, chronic comorbidities, and reduced access to healthcare, the association between race and negative clinical outcomes is often fraught with residual confounders [18,19,20]. The purpose of this review is to summarize the current state of the literature on these factors contributing to racial disparities in sepsis and to suggest potentially efficacious interventions. Specifically, we will be focusing on the impact of racial disparities on long-term outcomes since sepsis results in significant morbidity and mortality several years after the initial insult [21,22]. This review will also address the factors contributing to racial disparities under the framework of patient, community, and hospital-based factors [11,23].

## 2. Definitions of Race

Although racial differences in health outcomes have been studied throughout the past several decades, our understanding of race is still an evolving concept. However, it is important to define the racial populations that are commonly analyzed in epidemiologic studies on sepsis. These will be the definitions applied to later sections. In 1977, The Office of Management and Budget (OMB) offered a standardized classification of race for the purpose of census and subsequently revised the classification in 1997 [24]. According to the 1997 revision, “blacks” or “African-Americans” are defined as people having origins in any black racial group in Africa. “Hispanics” are defined as a people of Cuban, Mexican, Puerto Rican, Cuban, South or Central American, or any other Spanish culture. “Whites” are defined as people having origins in any of the original people of Europe, North Africa, or the Middle East [25]. Finally, “minority” race refers to any race (black, Hispanic, and others not discussed in this article) that is non-white.

## 3. Patient-Based Factors

### 3.1. Genetic Susceptibility

There is a growing body of literature discussing genetic determinants as a cause of increased susceptibility to infection and sepsis [2,8,15,18,23,26]. Single nucleotide polymorphisms (SNPs) have garnered much interest as a potential diagnostic and prognostic tool for septic patients. Many of the gene SNPs associated with an increased predisposition to sepsis are involved in vital cellular functions such as pathogen recognition, phagocytosis, and cytokine production [26,27]. Polymorphisms in the toll-like receptor (*TLR*) 1 gene, an important component of the innate immune response to pathogens, are associated with increased organ dysfunction and mortality in sepsis. This association persisted when evaluating white and black patients separately [28].

Gene SNPs can also confer a decreased susceptibility and survival benefit against sepsis and its complications [26]. A recent study by Rautanen and colleagues found that common variants in the *FER* gene, a non-transmembrane receptor tyrosine kinase involved in cell–cell adhesion, was associated with a significant decrease in 28-day mortality from pneumonia-derived sepsis. One *FER* allele, SNP rs4957796, was associated with a 44% reduction in 28-day mortality for each allele present. However, this study utilized a cohort of white European patients, which limits external validity to other patient demographics [29]. Another study by Ferwerda and colleagues found that an SNP within the *Mal* gene, an adaptor protein downstream from the TLR pathway, conferred resistance to septic shock. Compared across several populations, the authors found the allele to be in much higher frequency in West Eurasian populations compared to African [30].

In the age of genomic medicine, it appears that minority populations are among the last to benefit from its advances. Countless studies have provided genetic insights into conditions that predispose patients to sepsis such as diabetes and renal disease, but virtually all recent genomic studies utilized patients of European descent. This limits their generalizability to populations that are disproportionately affected by these conditions [23,31,32]. Currently no study has correlated genomic data with long-term outcomes beyond 90-day mortality [26,27,29,30,33].

While genetic susceptibility influences racial disparities in sepsis outcomes, social and environmental factors likely play a more significant role [11,13,32].

### 3.2. Presence of Comorbidities

Chronic conditions such as diabetes, renal failure, chronic obstructive pulmonary disease, obesity, and Human Immunodeficiency Virus infection (HIV) all place patients at an increased susceptibility to infection and sepsis. Preexisting comorbidities are also associated with an increased severity of disease and risk of hospital readmission following the initial event [3,5,6,7]. Racial disparities exist across many of these conditions with blacks experiencing both an increased prevalence and reduction in life expectancy compared to whites [13,34]. Blacks also possess more chronic comorbidities that alter immune function such as diabetes, HIV, and renal failure, which is associated with a greater risk of acute organ dysfunction and a relatively worse clinical course [15,16,35].

HIV infection is of particular importance since sepsis is one of the most common causes of mortality in these patients. One study found that 11.98% of black sepsis patients had an HIV-positive status compared to 0.69% of white sepsis patients [16]. The effect of HIV infection on long-term outcomes following sepsis is conflicting. A single-institution study in Brazil found severe sepsis to be an independent predictor of both 28-day mortality (adjusted hazard ratio (HR) of 3.13) and 6-month mortality (adjusted HR of 3.25) [36]. However, a recent study conducted at two European institutions found no significant difference in sepsis mortality between HIV-positive (50% mortality) and HIV-negative (42.8% mortality) up to 1 year following initial hospitalization [37].

Data regarding the relationship between gender and outcome in sepsis is relatively limited. A past study by Eachempati and colleagues found that gender was an independent predictor of mortality in critically ill octogenarian patients with sepsis [38]. There does not appear to be a significant difference in the incidence or rate of hospitalization based on gender, and these rates are similar across races in women [16]. In terms of management, a recent retrospective study found that there were no gender disparities in the sepsis bundle completion and in-hospital mortality [39].

### 3.3. Socioeconomic Status

Socioeconomic status (SES) is a very strong predictor of health outcomes in the United States [12,32,34]. Lower SES and minority groups are associated with lower rates of health insurance, decreased access to preventive health services, and poor health habits that contribute to inferior health outcomes [23,34]. Lack of insurance is independently associated with more severe clinical presentation, increased mortality, and lower utilization of acute care hospitalization following sepsis [16,40,41,42,43]. This suggests that uninsured patients often delay hospital presentation due to cost and receive lower intensity of medical treatment [41]. Key studies are summarized in Table 1.

Communities with higher rates of poverty, an indicator of lower SES, are associated with worse outcomes in sepsis [42,44]. Although minority populations are more likely to reside in communities with poverty, blacks still have a higher incidence of severe sepsis when adjusted for SES. Interestingly, Hispanic populations were found to have a slightly lower incidence after adjusting for urbanicity and poverty with an adjusted rate ratio of 0.91 compared to whites [44]. A similar finding was described in the context of post-surgical complications. The authors posited that family structures in Hispanics may be more conducive to an effective recovery and health promotion compared to other racial groups [45].

Sepsis often leads to additional comorbidities, and functional impairments following hospitalization and readmissions are common [5,6,7]. A recent study estimated the cost to be greater than 30,000 USD per sepsis-related readmission [5]. Since minority populations are more likely to be uninsured, they are forced to assume a greater share of the cost of care [40]. This only further hinders social mobility in this population and worsens their economic outlook as a greater share of disposable income is allocated to medical expenses. Since managing functional limitations and comorbidities is vital to reducing readmissions and mortality, these factors likely contribute to adverse long-term outcomes in septic patients [21,22,46].

## 4. Community-Based Factors

### 4.1. Access to Health Care

Differences in accessibility to health care such as preventive health services, primary care, and behaviors that promote health has been identified as a contributing factor to racial health disparities. Minority populations have limited access to healthy food options, exercise facilities, health information, safe environments, and quality healthcare [19,34,47,48].

Increased rates of obesity in blacks is frequently attributed to lack of access to healthy and affordable food options, while foods rich in fats are commonly available in these areas [47]. This pattern of diet characterized by added fats, fried foods, organ meats, and sugar-sweetened beverages has been associated with an increased risk of sepsis among blacks (adjusted HR of 1.41) [49]. Minority populations also exhibit higher rates of leisure-time physical inactivity, but these differences are eliminated when adjusted for education level [48]. This affirms several other studies noting educational achievement as a social determinant highly associated with positive health behaviors and outcomes [13,19,48].

### 4.2. Preventive Health Behaviors

Since many diseases are avoidable through lifestyle modification and access to primary care services, preventive health is at the forefront of health promotion initiatives. However, these efforts fall short in reducing health disparities for minority and disadvantaged populations. There is substantial evidence that minority populations have less access to primary care facilities and preventive health services [18,19,23]. One study found that communities with higher proportions of blacks were 28 times more likely to have lower access to primary care, even after adjustment for demographics and insurance status [50]. 

Pneumococcal vaccination (PCV-13 and PPSV-23) effectively reduces the incidence of pneumonia and sepsis [15,51,52]. Vaccination rates are consistently lower in minority groups and uninsured patients [34,51]. Also, patients who have a usual place for health care such as a community health center or outpatient primary care facility are more likely to be vaccinated than those who did not, regardless of insurance status [51,53]. Minority nursing home patients, who benefit most from vaccination, receive vaccinations at a rate of up to 15% less than white patients [54]. Substandard vaccination among elderly minority population places them at substantial risk for recurrent sepsis and adverse long-term outcomes.

### 4.3. Environmental Factors

The role of environmental factors such as geographic location, education, employment, and SES indicators have been associated with differences in susceptibility to disease [13,18,19,34,35,55]. Moore and colleagues described three regions in the southern United States that experienced significantly increased age-adjusted sepsis mortality rates from 2003 to 2012 relative to the rest of the United States (93.1 vs. 59.6 deaths per 100,000 persons respectively). These regions were characterized by lower education, income, insurance coverage, and higher unemployment [55]. Residence in a medically-underserved region, which is often the case in rural settings, has also been found to be associated with significantly higher sepsis mortality rates compared to better served areas [56]. This suggests that there is a complex interaction of socioeconomic factors that results in differences in sepsis outcomes that may not be fully explained by race alone.

Racism is a well-studied factor contributing to disparities in healthcare, but there is currently no literature describing racism in sepsis management [11,19,23]. Significant racial differences have been observed in the emergency department (ED) setting, which is where minority patients with sepsis commonly present [16,57,58]. One study found that black patients presenting to the ED received lower acuity ratings and experience significantly longer wait times following triage compared to white patients. For example, black patients waited an average of 95.6 and 98.3 minutes for chest pain and dyspnea respectively compared to 74.6 and 74.9 minutes in white patients [57]. This suggests that racial differences in the early identification and management of sepsis may be partially explained by the ED triage process.

Implicit bias, or the unconscious association of negative attitudes to individuals based on irrelevant characteristics such as race or gender, has been observed among healthcare professionals [59]. However, there are currently no studies that studied the role of implicit bias in the identification and management of sepsis. Implicit bias among physicians has been shown to diminish relationships with black patients based on patient-reported satisfaction surveys [60]. Racism also is a known contributor to residential segregation in the United States, creating areas with a high proportion of non-whites lacking access to medical care [11,19,50]. All these conditions may foster distrust of the medical profession within minority communities, which may limit the success of health promotion initiatives [11,19,60,61].

## 5. Hospital-Based Factors

### 5.1. Quality of Care

“Quality” in sepsis management is defined as adherence to clinical guidelines and outcome measures such as mortality and readmission rate [9,10]. Several studies have suggested that inter-hospital differences play a significant role in racial disparities of sepsis management. Minority patients are more likely to be treated at safety net hospitals and urban teaching hospitals, which often experience increased financial constraints while treating diverse patient populations [18,23,44,62,63]. Mayr and colleagues found that hospitals that treated higher proportions of black patients were less likely to receive antibiotics within 4 hours for pneumonia (odds ratio (OR) of 0.72 relative to whites) [63]. This difference was also seen in sepsis, where hospitals treating predominately minority patients have increased rates of organ dysfunction and mortality [15,44]. Key studies relating to the quality of sepsis management is summarized in Table 2.

However, there is increasing evidence refuting claims that minority patients receive inferior care in sepsis [16,35,55,64,65,66,67]. Dombrovskiy and colleagues found that while blacks experienced higher population-based mortality, age-adjusted case fatality rate was similar to whites (26.15 vs. 25.88 per 100 sepsis hospitalizations respectively) [16]. A similar finding was shown in a large retrospective study of United States acute care hospitals. The authors suggested that much of the differences in mortality among races was explained by preexisting conditions [16,35]. Several studies have even demonstrated a relative survival advantage among minority patients in pneumonia and sepsis, providing a stark contrast to previous literature [55,64,65,66,67].

### 5.2. Intensity of Care

Common interventions in the management of sepsis include mechanical ventilation, vasopressor and antibiotic administration, and admission to the intensive care unit (ICU) [2,9]. Several previous studies found that among sepsis patients, blacks were more likely to be hospitalized, require ICU-level care during the hospitalization, and experience a longer length of stay [15,16,23,35]. However, the conclusions of more recent studies have been inconsistent with prior literature. A retrospective study of administrative claims from six states found that blacks had a significantly higher incidence of sepsis and were less likely to receive ICU care, but had similar adjusted case fatality rates compared to whites [44]. A similar study by Moore and colleagues found that blacks had a lower incidence of sepsis and received similar intensity of care compared to whites [55]. An advantage to this study is that comprehensive chart review was employed for defining sepsis events, which is often more sensitive than administrative claims data [3,18,55].

Insurance status is a highly important predictor of health outcomes in critical illness, and appears to be a factor that limits the intensity of care in sepsis [18,19,23]. The association between black patients and lack of medical insurance or subsidized federal insurance such as Medicaid has been established [16,19,41,63,66]. Uninsured patients are more likely to delay necessary medical care, which results in increased organ dysfunction on presentation for sepsis [40,67]. Among patients admitted to the ICU for sepsis, uninsured patients experience a shorter length of stay, receive less interventions, and have inferior clinical outcomes compared to insured patients [40,42]. Lacking insurance is also associated with greater use of low-quality hospitals that fail to deliver guideline-adherent therapy [63,68,69].

### 5.3. Post-Acute Care Facility Utilization

With increasing hospitalizations and decreasing case fatality rates, there is a growing number of severe sepsis survivors with complex medical needs [21,22,46,70]. Approximately 25% of elderly patients hospitalized for sepsis are discharged to post-acute care facilities such as a skilled nursing facility (SNF) or long-term acute care hospital (LTACH) [7]. Non-white patients with sepsis are more likely to be discharged to home instead of an LTACH [14,35,41]. However, racial differences in post-acute care disposition was eliminated after controlling for insurance status [41]. This suggests that insurance status plays a more significant role in post-acute care utilization, and this has been supported in previous literature [40,41].

While this difference could be attributed to bias, it is more likely caused by factors differences in patient demographics and preferences for care [23]. Nonwhite sepsis patients, while more often presenting with increased organ dysfunction, are younger and could make a full recovery [14,16,23,55]. Black patients are also less likely to receive do-not-resuscitate orders in the ICU, so they more frequently undergo life-sustaining treatments during hospitalization regardless of previous comorbidities or functional status [71,72]. 

Discharge to a care facility has been frequently associated with increased likelihood of readmission among sepsis survivors [5,6,7,73]. Recurrent infection sepsis is one of the most common causes of readmission in this population and is associated with poor long-term outcomes and mortality [22,73]. There is also a great deal of variability in care delivered at SNFs in regards to readmission and mortality rates [54,73]. More research is needed to understand the factors that drive increased readmissions and poor outcomes at post-acute care facilities.

## 6. Interventions to Reduce Racial Disparities in Sepsis

Racial disparities are frequently described, but interventions to reduce such disparities are sparse [18,23,34]. While there have been several studies describing the implementation of quality improvement programs in sepsis management, there are currently no studies that report reductions in racial disparity as an outcome [23,74,75,76,77].

Patient-based interventions should address the lack of inclusion of minority patients in genomic studies of sepsis. This population offers an exciting opportunity for researchers to uncover genomic insights into social determinants of heath [31,32]. Future research efforts should also focus on the impact of quality initiatives on reducing disparities in the management of chronic comorbidities such as diabetes and chronic kidney disease [23,78,79]. 

Community-based interventions should focus on increasing access to primary care health services in lower SES neighborhoods. This could include the expansion of community health centers and mitigating barriers to healthcare access such as lack of availability of services and the financial burden of care [11,23,80]. To better elucidate geographic disparities in healthcare, methods such as county-level health statistics and census studies have shown promise and should continue to be utilized [42,50]. Public policy efforts should also be directed towards uniform vaccination coverage, especially among susceptible populations such as minority nursing home residents [51,54,73]. Finally, further research is needed to understand the impact of implicit bias and racism among healthcare providers on processes of care for minority populations [11,19,23,61].

Hospital-based interventions should aim at reducing inter-hospital and intra-hospital variation in clinical management. This includes systematically analyzing physician adherence to treatment protocols and establishing uniform admission to post-acute care facilities [22,23,77]. Administrative efforts should also explore strategies to mitigate the effects of insurance status as a source for disparities in sepsis management [40,41].

Finally, further research should aim to develop more sophisticated statistical methods to address factors that confound the relationship between race and outcomes in sepsis. Race has been shown to be associated with differences relating to access to health care, preventive health services, and health literacy. It is very unlikely that these differences are being adequately controlled in statistical analysis, so it is important to continue to develop methods to address variables with complex implications on health outcomes.

## 7. Conclusions

Sepsis is a common condition associated with significant morbidity and mortality. The literature describes racial disparities in the management of sepsis with several underlying factors contributing to the differences in patient outcomes. Only through addressing the complex interaction between race and these factors can policy makers enact quality improvement initiatives that effectively addresses these disparities.

## Figures and Tables

**Table 1:** Selected studies on the association of socioeconomic status (SES), race, and sepsis outcomes.

Reference	Data Source	Years	Findings
[40]	Nationwide sample	2000–2008	Uninsured patients were more likely to be younger, male, non-white, and lived in lower income ZIP codes than insured patients Uninsured patients had fewer comorbidities than insured patients, but were more likely to have cirrhosis or HIV infection Uninsured patients had a higher adjusted mortality rate than those with private insurance (OR, 1.43; 95% CI, 1.37–1.47)
[41]	Pennsylvania statewide sample	2004–2006	Less blacks have commercial insurance compared to whites (14.9% vs. 23.4%), but more likely to have Medicaid or no insurance. Medicaid patients are less likely to be discharged to long-term care facilities (OR, 0.17; 95% CI, 0.13–0.24)
[42]	U.S. county-level mortality data	2003–2012	Counties with higher rates of sepsis mortality were associated with higher percentage of black residents (13.4% vs. 1.7%) and lower educational achievement (12.8% vs. 17.2% completing college)
[43]	California statewide sample	2010	Lack of insurance was associated with a higher risk of organ dysfunction on presentation (adjusted OR, 1.26) Lack of insurance predicted in-hospital mortality (adjusted OR, 1.15)

Odds ratio (OR), confidence interval (CI).

**Table 2:** Selected studies on the quality of sepsis management among racial groups.

Reference	Data Source	Years	Findings
[63]	28 U.S. hospitals	2001–2004	Blacks less likely to receive antibiotics within 4 h (OR, 0.55; 95% CI, 0.43–0.70) and to receive guideline-adherent therapy (OR, 0.72; 95% CI, 0.46–0.76) Within the same hospital, blacks and whites received similar quality of care (OR, 1.0; 95% CI, 0.97–1.04)
[16]	New Jersey State Inpatient Database	2002	Blacks had greater rates of hospitalization in sepsis across all age groups with the greatest disparity at age 35–44 (RR, 4.35; 95% CI, 3.93–4.82) Blacks had similar case fatality rates compared to whites (26.15 ± 0.36 vs. 25.88 ± 0.79 in whites and blacks, respectively)
[35]	National Hospital Discharge Survey (NHDS)	1979–2003	Blacks and other races more likely to develop sepsis than whites (average black annual RR, 1.90; 95% CI,1.82–1.98; average annual other race RR, 1.85; 95% CI, 1.75–1.95) The median length of hospital stay was longer in blacks compared to whites and other races (1.0 day greater, 95% CI, 1.8–2.2) Case fatality rates were lower in other races (18%) compared to whites (21%) and blacks (21%)
[55]	Nationwide sample	2003–2007	Incidence of first-sepsis events lower among blacks (adjusted HR, 0.64; 95% CI, 0.57–0.72) Odds of sepsis similar between blacks and whites (adjusted OR, 1.01; 95% CI, 0.84–1.21) Blacks more likely to be diagnosed with severe sepsis among first-sepsis events (76.9% vs. 71.5%)
[64]	Veterans Health Administration (VHA) nationwide sample	2002–2007	Blacks and whites equally likely to receive guideline-concordant antibiotic therapy (adjusted OR, 0.98; 95% CI, 0.87–1.10) Blacks had a shorter hospital length of stay (adjusted OR, 0.84; 95% CI, 0.76–0.93) Blacks experienced a lower 30-day mortality (adjusted OR, 0.82; 95% CI, 0.68–0.99)
[65]	42-state sample of ~420 U.S. hospitals	2012–2014	Blacks were less likely to be hospitalized for sepsis (adjusted OR, 0.97; 95% CI, 0.96–0.98) Blacks had lower hospital mortality than whites (adjusted OR, 0.85; 95% CI, 0.84–0.86)
[67]	California statewide sample	2011	The unadjusted case fatality rate for blacks (14.0%) and Hispanics (13.8%) was less than whites (15.1%) (*p* < 0.001)

Odds ratio (OR), confidence interval (CI), relative risk (RR), hazard ratio (HR).
